# Functional outcomes after retrosigmoid approach to the cerebellopontine angle: Observations from a single-center experience of over 13 years

**DOI:** 10.1016/j.bas.2024.102909

**Published:** 2024-08-08

**Authors:** Amir Kaywan Aftahy, Maria Goldberg, Vicki M. Butenschoen, Arthur Wagner, Bernhard Meyer, Chiara Negwer

**Affiliations:** Technical University Munich, School of Medicine, Klinikum rechts der Isar, Department of Neurosurgery, Munich, Germany

**Keywords:** Retrosigmoid approach, Neurosurgery, Operative technique, Skull base surgery, Cerebellopontine angle

## Abstract

**Introduction:**

Accessing the posterior base of the skull is complex because of the vital neurovascular structures in the area. However, the retrosigmoid approach (RSA) offers a solution to this challenge.

**Research question:**

To analyze surgical outcome of RSA.

**Material and methods:**

This study involved a retrospective review of patient charts from a single center, focusing on the surgical procedure and outcomes following the operation.

**Results:**

The study included 517 patients suffering from conditions like vestibular schwannomas (VS), metastatic cancers, and trigeminal neuralgia. The most frequent symptoms reported were balance disorders (42.7%), hearing loss (36.5%), walking difficulties (21.2%), headaches (18.9%), facial pain (17.1%), issues with trigeminal nerve function (14.1%), cerebellar dysfunction (13.5%), and facial nerve paralysis (10.2%). The rate of complications stood at 21.1%, with 11.3% of patients needing revision surgery. The median score on the Clavien-Dindo scale was 2, and the rate of mortality related to surgery was 1.0%. Permanent symptom improvement was seen in 72.1% of cases. Temporary new deficits occurred in 43.2% of patients, with facial nerve paralysis being the most common (14.1%). No significant correlation was found between the size of the craniotomy and the extent of tumor resection (p = 0.155), except in the case of VS (p = 0.041). Larger craniotomy sizes were associated with higher rates of complications (p = 0.016), especially CSF leaks (p = 0.006). Complications significantly affected the likelihood and number of new deficits (p < 0.001 for both), particularly postoperative bleeding (p = 0.019, p = 0.001), CSF leaks (p = 0.026, p = 0.039), and hydrocephalus (p = 0.050, p = 0.007).

**Conclusions:**

The potential for complications related to the surgical approach cannot be overlooked. The size of the tumor should not dictate larger surgical approaches due to the associated increase in postoperative complications; a tailored approach that considers the precise tumor location and pathology is crucial for optimizing postoperative outcomes.

## Highlights

• Rectosigmoid approach allows access to the CPA, which is complex due to crucial neurovascular structures.
• Larger craniotomy is associated with higher complication rate including CSF leakage.
• Larger tumor size must not lead to larger approaches.

## 1 Introduction

The cerebellopontine angle (CPA) is associated with various pathologies such as chordomas, schwannomas, and neurovascular compression syndromes (Bambakidis et al., 2007; Rhoton, 2000; Samii et al., 2006, 2013; Sanna et al., 2010), extending from the inferior border of the internal auditory canal to the exit zone of the glossopharyngeal nerve. It is bounded anteriorly by the petrous portion of the internal carotid artery and medially by the petrous apex (Samii et al., 1995a, 1995b; Samii and Matthies, 1997).

Several surgical approaches to this area, such as the trans-petrous or the trans-cochlear approach, compromise hearing and endanger the facial nerve (Diaz Day, 2012; Samii et al., 2013). Therefore, the retrosigmoid approach has been deemed the workhorse of the posterior fossa and other CPA approaches. Technical details and variations have been discussed previously (Arnone et al., 2020; Elhammady et al., 2012; Elsmore and Mendoza, 2002; Ramina et al., 2008b; Samii et al., 1995b).

The purpose of this study was to share our experience with a large series of RSAs for various pathologies. This study sheds light on the associated complications, surgical success, and postoperative outcomes to improve effectiveness and reduce perioperative morbidity.

## 2 Materials and methods

### 2.1 Retrosigmoid approach (RSA)

Analogous to the pterional approach for the anterior skull base, RSA is the standard approach for CPA. The technical details have been previously discussed in detail (Elsmore and Mendoza, 2002; Ramina et al., 2008b; Samii et al., 1995b). A C-shaped skin incision was preferred. The main steps in RSA are as follows: an osteoplastic or osteoclastic approach is chosen, with the superior and anterior margins adjacent to the transverse and sigmoid sinuses, respectively. The initial release of CSF from the cerebellomedullary cistern is recommended to expose the CPA. The two layers of the arachnoid are separated, and the lesion/pathology is markedly decompressed before dissection. The posterior wall of the internal auditory canal is drilled if necessary.

### 2.2 Study population and clinical parameters

This was a non-interventional, retrospective, monocentric study conducted by the Department of Neurosurgery of Klinikum rechts der Isar. Clinical documentation, neuropathological records, and corresponding pre- and postoperative imaging of patients aged 18 years and older who had undergone skull base surgery via the RSA between January 2007 and September 2020 were examined.

Patient-related data collected included the sex and age of the patients at the time of surgery. To include general health risk factors, information on the consumption of nicotine, alcohol, and drugs, the use of blood-thinning and immunosuppressive drugs, and the presence of a previous disease significant to the operation or healing process were included. The Karnofsky Performance Status Scale (KPSS) scores and preoperative neurological symptoms were recorded. Preoperative symptoms included cranial nerve-related symptoms. Facial nerve palsy was graded using the House-Brackmann classification. Given that not all patients underwent tone audiometric examination, patients with VS requiring surgery were dichotomized as having useful or non-useful hearing loss pre- or postoperatively (the ability to use the telephone on the affected side). The use of anticonvulsants and/or analgesics was recorded for patients with trigeminal neuralgia. As part of the data collection, the tumor entity, tumor volume, affected side, presence of initial diagnosis or recurrence, and the World Health Organization grade were considered for oncologic patients. In addition, if the tumor was recurrent, prior treatment(s) documentation was also done if radiotherapy, chemotherapy, and/or surgical resection had already been performed prior to surgery.

Metastases resected via RSA during the defined period were classified according to their primary malignancies and according to their occurrence as solitary, singular, or multiple. Radiological outcome parameters consisted of the anatomic location as well as the extent of resection (EOR), defined by comparing the preoperative and postoperative 3.0 T cranial magnetic resonance imaging (MRI). This was achieved using T1 ± contrast agent sequences by manual volumetric segmentation using the Origin® software (Brainlab, version 3.1, BrainlabAG, Munich, Germany). We used the patients’ existing MRI scans for craniotomy measurement. To obtain a three-dimensional impression of the craniotomy defect, the defect was first marked as precisely as possible in two views—axial and coronal—using crosshairs consisting of two planes. In the sagittal view, the defect appears as approximately three-dimensional in one impression, which a region of interest (ROI) area measurement tool could determine in the largest diameter.

Meningiomas and VS were subdivided according to the classification of Desgeorges and Sterkers and Yasargil and the Hannover classification, respectively (Qin et al., 2021; Samii and Matthies, 1997).

The postoperative data included the degree of resection, postoperative KPSS score, complications with any necessary revisions, new permanent neurological deficits, and improvement in preoperative neurological symptoms.

In addition, the worsening of preoperative symptoms, length of hospital stay in days, and follow-up time in months were recorded.

New postoperative deficits were defined as permanent if they persisted until the last follow-up examination at the time of evaluation.

The degree of resection is also referred to as gross total resection (GTR) in cases where tumor removal is confirmed using MRI. For meningiomas, the degree of resection is described by the corresponding Simpson grade. Tumors with Simpson grades I and II, or GTR, were defined as completely resected tumors.

Postoperative complications, reinterventions, and adverse events were collected and summarized according to the Clavien-Dindo Scale (CDC). The postoperative complications included rebleeding, cerebrospinal fluid leaks or fistulas, wound infections/healing disorders, sinus venous thrombosis (SVT), intracranial abscesses, meningitis, ischemia, and hydrocephalus.

Statistical analyses, including descriptive data analyses, were performed using the IBM SPSS Statistics Version 26.0 (SPSS Inc.; IBM Corp., Armonk, NY, USA). Data are shown as median and interquartile range or mean and standard deviation. Spearman's non-parametric rank correlation test was used to examine the relationships between continuous variables. For categorical variables, unpaired Mann–Whitney U tests were used to compare the two samples. Proportions and group differences were analyzed using the chi-square test or Fisher's test if the sample size was insufficient. P-value ≤0.05 was considered significant.

This study was approved by the local ethics committee of the Technical University of Munich School of Medicine (231/20 S-EB). The requirement for written informed consent was waived by the ethics committee. This study was conducted according to the ethical standards of the 1964 Declaration of Helsinki and its later amendments.

## 3 Results

### 3.1 Study population

Between January 2007 and September 2020, 540 procedures were performed in 517 patients aged 19–90 years. A total of 204 (37.8%) procedures were performed on male patients and 336 (62.2%) on female patients. Surgery was performed for heterogeneous pathologies. Among them were 180 VS, 14 non-VS (trigeminal, glossopharyngeal, vagal and accessory shwannomas), 157 meningiomas, 71 cases of trigeminal neuralgia, nine cases of hemifacial spasm, 49 metastases, 15 epidermoid cysts, nine hemangiomas, and 36 other cases (3 anaplastic astrocytomas, one arachnoid cyst, five non-Hodgkin B-cell lymphomas, four intracranial abscesses due to transmigrated otitis media, one ependymoma, two glomus jugular tumors, two glossopharyngeal neuralgias, five cavernous vascular malformations, three cerebellar radiation necroses, one bone hemangioma, one malignant glioma, two medulloblastomas, one subependymoma, one microvascular compression of the vestibular nerve, one plexus papilloma, one reactive astrogliosis, and two diffuse midline gliomas) localized in the cerebellopontine angle or adjacent structures of the posterior skull base. The most common neurological symptoms were dizziness and/or balance disorders in 42.7% of the cases, gait unsteadiness in 21.2%, headache in 18.9%, and cerebellar dysfunction in 13.5% of the patients. Among cranial nerve-related preoperative symptoms, hearing impairment (36.5%) was the most common symptom, followed by facial pain and trigeminal dysfunction such as hypo-, par-, and dysesthesia (31.2%), tinnitus (13.7%), and facial nerve palsy in 10.2% of cases. Nearly half of the patients (42.5%) had been suffering from symptoms for over a year (Table 1).

**Table 1:** Clinical characteristics.

Parameter N (%)	Epidermoid cyst (N = 15)	Hemangioblastoma (N = 9)	Hemifacial spasm (N = 9)	Meningioma (N = 157)	Metastasis (N = 49)	Non-vestibular schwannoma (N = 14)	Other (N = 36)	Trigeminal neuralgia (N = 71)	Vestibular schwannoma (N = 180)
Age mean SD	50.2 ± 11.8	49.3 ± 19.3	56 ± 13.8	60.3 ± 14.1	62.7 ± 13.4	56.4 ± 18.3	51.4 ± 17.9	61.9 ± 13.8	54.5 ± 15.1
Male	8 (53.3%)	5 (55.6%)	4 (44.4%)	39 (24.8%)	20 (40.8%)	7 (50%)	18 (50%)	29 (40.8%)	74/41.1%)
Female	7 (46.7%)	4 (44.4%)	5 (55.6%)	118 (75.2%)	29 (59.2%)	7 (50%)	18 (50%)	42 (59.2%)	106 (58.9%)
cardiovascular disease	3 (21.4%)	4 (44.4%)	4 (66.7%)	74 (50.3%)	17 (50.3%)	8 (61.5%)	10 (28.6%)	33 (47.1%)	62 (37.6%)
oncological disease	2 (14.3%)	3 (33.3%)	0	32 (21.8%)	48 (21.8%)	4 (30.8%)	10 (28.6%)	11 (15.7%)	28 (17.0%)
coagulation disorder	0	0	0	8 (5.4%)	3 (5.4%)	0	0	1 (1.4%)	5 (3.0%)
metabolic disease	21,4%	1 (11.1%)	2 (33.3%)	31 (21.1%)	7 (21.1%)	4 (30.8%)	2 (5.7%)	10 (14.3%)	18 (10.9%)
nicotine/alcohol/drug abuse	21,4%	1 (11.1%)	1 (16.7%)	19 (13.1%)	4 (13.1%)	0	3 (8.8%)	14 (20.3%)	18 (10.9%)
Preoperative symptoms									
no symptoms	2 (13.3%)	0	0,0%	17 (10.8%)	4 (8.3%)	0	2 (5.6%)	0	5 (2.8%)
headache	4 (26.7%)	6 (66.7%)	1 (11.1%)	33 (21.0%)	21 (43.8%)	2 (14.3%)	10 (27.8%)	3 (4.2%)	22 (12.2%)
vertigo	8 (53.3%)	4 (44.4%)	1 (11.1%)	69 (43.9%)	31 (64.6%)	5 (35.7%)	12 (33.3%)	3 (4.2%)	97 (53.9%)
nausea/emesis	2 (13.3%)	1 (11.1%)	1 (11.1%)	12 (7.6%)	18 (37.5%)	2 (14.3%)	6 (16.7%)	0	14 (7.8%)
cerebellar dysfunction	0	1 (11.1%)	0,0%	22 (14.0%)	22 (45.8%)	2 (14.3%)	12 (33.3%)	0	14 (7.8%)
gait disturbance	4 (26.7%)	1 (11.1%)	0,0%	43 (27.4%)	16 (33.3%)	2 (14.3%)	7 (19.4%)	2 (2.8%)	39 (21.7%)
hydrocephalus	1 (6.7%)	0	0,0%	18 (11.5%)	9 (18.4%)	0	7 (19.4%)	0	10 (5.6%)
visual impairment	1 (6.7%)	0	0,0%	7 (4.5%)	1 (2.1%)	1 (7.1%)	1 (2.8%)	0	2 (1.1%)
hemiparesis	0	0	0,0%	4 (2.5%)	0	0	2 (5.6%)	0	2 (1.1%)
vigilance reduction	0	0	0,0%	4 (2.5%)	1 (2.1%)	0	3 (8.3%)	0	2 (1.1%)
psychomotoric disturbance	0	0	0,0%	5 (3.2%)	5 (10.4%)	1 (7.1%)	5 (13.9%)	0	1 (0.6%)
seizure	1 (6.7%)	0	0,0%	4 (2.5%)	2 (4.2%)	0	0	0	0
trigeminal hypesthesia V1	2 (13.3%)	0	1 (11.1%)	10 (6.4%)	0	1 (7.1%)	2 (5.6%)	2 (2.8%)	14 (7.8%)
trigeminal hypesthesia V2	4 (26.7%)	0	1 (11.1%)	15 (9.6%)	1 (2.1%)	4 (28.6%)	4 (11.1%)	15 (21.1%)	24 (13.3%)
trigeminal hypesthesia V3	3 (20.0%)	0	1 (11.1%)	10 (6.4%)	0	3 (21.4%)	4 (11.1%)	7 (9.9%)	23 (12.8%)
trigeminal hypesthesia total	5 (33.3%)	0	1 (11.1%)	17 (10.8%)	1 (2.1%)	4 (28.6%)	5 (13.9%)	16 (22.5%)	29 (16.1%)
facial pain	0	0	1 (11.1%)	15 (9.6%)	0	1 (7.1%)	2 (5.6%)	15 (21.1%)	2 (1.1%)
ocular motility disorder	3 (20.0%)	0	0	13 (8.3%)	0	0	5 (13.9%)	0	2 (1.1%)
facial nerve palsy (HB-grade 2)	0	0	0	5 (3.2%)	1 (2.1%)	1 (7.1%)	2 (5.6%)	0	18 (10.0%)
facial nerve palsy (HB-grade 3)	0	0	11,1%	2 (1.3%)	2 (4.2%)	1 (7.1%)	2 (5.6%)	1 (1.4%)	7 (3.9%)
facial nerve palsy (HB-grade 4)	1 (6.7%)	0	0	1 (0.6%)	1 (2.1%)	0	0	0	4 (2.2%)
facial nerve palsy (HB-grade 5)	0	0	0	0	0	1 (7.1%)	0	0	0
facial nerve palsy (HB-Grad unknown)	0,0%	0	0	1 (0.6%)	1 (2.1%)	0,0%	0	1 (1.4%)	0
facial nerve palsy total	1 (6.7%)	0	0	9 (5.7%)	5 (10.4%)	3 (21.4%)	4 (11.1%)	2 (2.8%)	29 (16.1%)
facial spasm	1 (6.7%)	0	0	1 (0.6%)	0	0	2 (5.6%)	3 (4.2%)	4 (2.2%)
hearing loss	4 (26.7%)	11,1%	1 (11.1%)	28 (17.8%)	4 (8.3%)	6 (42.9%)	6 (16.7%)	0	147 (81.7%)
tinnitus	5 (33.3%)	1 (11.1%)		11 (7.0%)	2 (4.2%)	2 (14.3%)	2 (5.6%)	1 (1.4%)	50 (27.8%)
CN IX/X	0	0	0	16 (10.2%)	1 (2.1%)	0	2 (5.6%)	0	3 (1.7%)
CN XII	0	0	1 (11.1%)	4 (2.5%)	0	2 (14.3%)	0	0	2 (1.1%)

In 63 (12.6%) cases, patients reported regular use of nicotine or alcohol. In 77 (15.5%) cases, patients reported taking blood-thinning medications, and 26 (5.2%) cases, immunosuppressive medications. Approximately 215 (43.3%) patients had a previous cardiovascular disease at the time of surgery, 138 (27.2%) had a known oncological disease, 14 (2.8%) had a blood coagulation disorder, and 78 (15.4%) had a metabolic disease. The median KPSS was 90%.

A significant association was found between the presence of a coagulation disorder (e.gt. thrombophilia) and the occurrence of postoperative SVT (p = 0.032). Other thromboembolic events, such as the postoperative occurrence of pulmonary artery embolism or deep vein thrombosis, did not show this association (p = 1.00). The presence of metabolic disease did not show a statistically significant influence on the occurrence of postoperative complications, such as wound healing disorders or wound infections (p = 0.121), intracranial abscesses (p = 0.223), or a history of nicotine, alcohol, or drug consumption (p = 0.196; p = 0.579). Immunosuppressive medications (p = 1.00) and preoperative radiotherapy (p = 0.434) did not result in significant postoperative complications. These results are consistent with those of the isolated analysis of the oncology-only and non-oncology subgroups.

### 3.2 Tumor-related data

The median volume of tumors operated via RSA was 5.78 cm^3^ (range 0.06–99.1). Among the operated metastases, the most common site of origin of these metastases was lung cancer (24.5%), followed by breast cancer (20.4%) and malignant melanoma (12.2%). The localization of meningiomas, according to Desgeorges and Sterkers, and Yasargil, is shown in Table 2. Seventy-five (42.6%) cases of the VS may be assigned to Hannover class T4a or b. Syndromic association was found in 12 cases (2.2%), neurofibromatosis type 2 (NF2) was detected in eight, and Von Hippel-Lindau syndrome in four cases.

**Table 2:** Anatomical localization of meningiomas.

Meningioma	N (%)
Petroclival	56 (36.8%)
Petrous	68 (44.8%)
Tentorial	28 (18.4%)
Petrous meningiomas according to Desgeorges and Sterkers	
anterior	9 (14.1%)
medial	17 (26.6%)
posterior	17 (26.6%)
medial and anterior	12 (18.8%)
medial and posterior	8 (12.5%)
anterior, medial, and posterior	1 (1.6%)
Tentorial meningiomas according to Yasargil	
T1-T2	5 (18.5%)
T6-T7	22 (81.5%)

Meningiomas, non-VS, and VS were found to be associated with NF2 in 2.3% (8/351) of the cases, and hemangioblastomas were associated with Von Hippel-Lindau syndrome in 44.4% (4/9).

The occurrence of neurological deficits correlated with tumor size (p < 0.001). In contrast, complete resection could not be statistically related to tumor volume (p = 0.067), but the exceptions included meningiomas and VS. The larger the meningioma, the more likely it was that only a resection grade of Simpson III or IV was achieved (p = 0.001), which can be considered a result of the function-remaining resection. In addition, for VS, a large tumor volume tended to contradict the achievement of GTR (p = 0.024). Regarding the postoperative complication rate, there was no correlation with tumor volume (p = 0.086); however, a more frequent occurrence of postoperative hemorrhage (p = 0.035) and hydrocephalus (p = 0.019) was observed in the presence of larger tumors. A larger tumor volume was associated with larger craniotomies (p = 0.009).

### 3.3 Approach-related findings and technical details

The median craniotomy defect size was 771 mm. Osteoclastic craniotomies were performed in 66.1% (355) and osteoplastic craniotomy in 33.9% (182) of the cases. For the reconstruction of the bony defect, the surgeons opted for bone meal in 57.2% of the cases and for the replacement of the bone flap in 31.1%. In 9.3% of the cases, closure was performed using a titanium mesh, and in 2.3%, bone cement was used. The intraoperative extent of the craniotomy was necessary in 3.2% of the cases.

Table 3 provides an overview of the postoperative complications. Reconstruction using titanium meshes showed a statistically higher incidence of postoperative complications (p = 0.020); however, the type of craniotomy did not correlate with the postoperative complications (p = 0.131).

**Table 3:** Postoperative complications.

Complication N (%)	Epidermoid cyst (N = 15)	Hemangioblastoma (N = 9)	Hemifacial spasm (N = 9)	Meningioma (N = 157)	Metastasis (N = 49)	Non-vestibular schwannoma (N = 14)	Other (N = 36)	Trigeminal neuralgia (N = 71)	Vestibular schwannoma (N = 180)
**Postoperative complications**	4 (28.6%)	1 (11.1%)	1 (11.1%)	32 (22.4%)	9 (21.4%)	4 (28.6%)	7 (21.9%)	9 (13.0%)	102 (21.1%)
**hemorrhage**	0	0	0	6 (4.2%)	0	0	0	1 (1.4%)	13 (2.7%)
**CSF leak**	1 (7.1%)	0	0	8 (5.6%)	4 (9.5%)	2 (15.4%)	1 (3.1%)	2 (2.9%)	36 (7.5%)
**wound healing disorder**	1 (7.1%)	0	0	5 (3.5%)	4 (9.5%)	0	2 (6.3%)	3 (4.3%)	22 (4.6%)
**intracranial abscess**	0	0	0	1 (0.7%)	4 (9.5%)	0	1 (3.1%)	1 (1.4%)	6 (1.2%)
**hydrocephalus**	0	0	0	7 (4.9%)	2 (4.8%)	2 (15.4%)	3 (9.4%)	1 (1.4%)	18 (3.7%)
**SVT**	1 (7.1%)	0	1 (11.1%)	6 (4.2%)	1 (2.4%)	0	0	0	11 (2.3%)
**meningitis**	1 (7.1%)	0	0	10 (7.0%)	0	0	1 (3.1%)	3 (4.3%)	24 (5.0%)
**ischemia**	0	1 (11.1%)	0	4 (2.8%)	0	0	2 (6.3%)	0	10 (2.1%)
**death**	0	0	0	3 (2.1%)	2 (4.8%)	0	0	0	5 (1.0%)
**revision surgery**	**3 (20.0%)**	**0**	**0**	**21 (13.4%)**	**11 (22.4%)**	**4 (28.6%)**	**8 (22.2%)**	**5 (7.0%)**	**80 (14.8%)**
**CSF leak**	0	0	0	6 (3.8%)	4 (8.2%)	0	1 (2.8%)	2 (2.8%)	26 (4.8%)
**wound revision**	1 (6.7%)	0	0	3 (1.9%)	4 (8.2%)	0	0	2 (2.8%)	15 (2.8%)
**abscess evacuation**	0	0	0	0	2 (4.1%)	0	1 (2.8%)	0	4 (0.7%)
**hematoma evacuation**	0	0	0	4 (2.5%)	0	0	0	1 (1.4%)	10 (1.9%)
**EVD**	0	0	0	9 (5.7%)	1 (2.0%)	1 (7.1%)	2 (5.6%)	1 (1.4%)	17 (3.1%)
**VP Shunt**	0	0	0	6 (3.8%)	0	2 (14.3%)	2 (5.6%)	1 (1.4%)	14 (2.6%)
**CDC I**	0	1 (100%)	1 (100%)	6 (18.8%)	0	3 (75%)	0	1 (12.5%)	16 (18.0%)
**CDC II**	3 (100%)	0	0	12 (37.5%)	3 (50.0%)	1 (25%)	0	3 (37.5%)	33 (37.1%)
**CDC IIIa**	0	0	0	1 (3.1%)	2 (33.3%)	0	0	0	4 (4.5%)
**CDC IIIb**	0	0	0	4 (12.5%)	1 (16.7%)	0	0	3 (37.5%)	19 (21.3%)
**CDC IV**	0	0	0	6 (18.8%)	0	0	3 (100%)	1 (12.5%)	12 (13.5%)
**CDC V**	0	0	0	3 (9.4%)	0	0	0	0	5 (5.6%)

(SVT) sinus venous thrombosis, (CSF) cerebrospinal fluid, (EVD) extraventricular drainage, (VP) ventriculoperitoneal shunt, (CDC) Clavien-Dindo classification.

Analysis of the craniotomy area showed no significant correlation with the extent of resection (p = 0.155). In the case of the VS, the size of the craniotomy played a role in the GTR (p = 0.041).

### 3.4 Postoperative and outcome-related data

The median hospital stay was nine days. The median follow-up period was ten months (range 0–150). The postoperative KPSS was 90%. The total complication rate was 21.1%, including CSF leaks, meningitis, postoperative bleeding, wound healing disorders and infections, hydrocephalus, ischemia, intracranial abscesses, and SVT. The median Clavien-Dindo score was 2. General postoperative complications, such as pneumonia or UTI, occurrence of pulmonary artery embolism or deep vein thrombosis, and occurrence of tympanic effusions, were also considered. Complete resection was achieved in 257 (57.2%) cases.

Common postoperative complications were CSF leaks or fistulas in 36 (7.5%) procedures, followed by meningitis in 24 (5.0%), and wound infections in 22 (4.6%) cases. Postoperative hydrocephalus occurred after 18 (3.7%) operations and required shunting in 3 cases (77.8%). The surgery-related mortality rate was 1% (five patients). Surgical revision due to these complications was performed in 61 (11.3%) cases. Postoperative recurrence occurred in 16 (4.7%), and postoperative progression of intraoperatively remaining tumors occurred in 46 (13.5%) cases during follow-up. Radiotherapy was performed in 109 (24.2%) patients and chemotherapy in 27 patients (5.8%). A repeat surgical procedure for tumor resection was subsequently performed in 30.6% of these recurrences/progressions during the follow-up period.

In 81.6% of cases, there was no deterioration of postoperative symptoms. In 72.1% of the patients, at least one preoperative symptom improved permanently after surgery. Patients who suffered from facial spasms due to hemifacial spasms reported sustained improvement in 100% of cases. In patients with trigeminal neuralgia, sustained improvement in facial pain was observed in 7 out of 10 patients. Additionally, 40.4% of the preoperative facial nerve palsies were alleviated or disappeared completely postoperatively. Postoperative improvement in hearing loss was achieved in 13.7% of cases. A postoperative deterioration of pre-existing facial nerve palsy was observed in 25.5% of the cases. Likewise, hearing deterioration on the affected side was found in 30.3% of the cases. In 11.7% of the patients with VS and preserved functional hearing, complete hearing loss occurred postoperatively.

After 221 (43.2%) surgeries, at least one new permanent neurological deficit occurred. Frequent neurological deficits included facial nerve palsy (72/14.1%), mainly with House-Brackmann grades 2 and 3, hearing loss (42/8.2%), dizziness and balance disorders (36/7.0%), and headaches (35/6.8%).

The presence of postoperative complications had a strong influence on the occurrence and number of permanent new deficits (p < 0.001 and p < 0.001, respectively), specifically the occurrence of postoperative hemorrhage (p = 0.019, p = 0.001), postoperative CSF leaks or fistulas (p = 0.026, p = 0.039), and postoperative hydrocephalus (p = 0.050, p = 0.007). CSF leaks and fistulas correlated particularly with the occurrence of dizziness and balance disorders as well as cerebellar dysfunctions as new deficits.

The age of the patients did not play a statistically significant role in the postoperative complication rate, improvement or worsening of symptoms, postoperative KPSS score, or the occurrence of new neurological deficits (p > 0.05).

## 4 Discussion

Publications regarding approaches to the posterior skull base and the CPA have increased with respect to the so-called “keyhole approaches” (Caballero-García et al., 2021; de Tribolet et al., 2011). There is a trend toward further reduction in approach size and invasiveness, presumably resulting in better postoperative outcomes (Ramina et al., 2008a).

Our results showed that larger craniotomy areas were associated with a more frequent occurrence of CSF leakage. Kehler et al. also reported a significant association between the size of the bony defect and the incidence of CSF fistulas in a prospective study using various approaches (Kehler et al., 2012). If a CSF leak occurred during the postoperative course, the patients in this study showed a statistically significant increase in the occurrence of new deficits.

Hoshide et al. postulated that the keyhole approach is not equivalent to a smaller craniotomy area. Rather, it is a concept of using preoperative image data sets and intraoperative navigation to plan the surgical approach as precisely as possible (Hoshide et al., 2018), implying that excessively large craniotomies can be avoided with sufficient preoperative planning. However, avoiding the intraoperative extent in this approach is essential. Neuronavigation may be an important tool for appropriate planning of the extent of craniotomy. Although it was not associated with an increased incidence of postoperative complications, additional intraoperative craniotomy should be avoided due to prolonged operation time and possibly more complicated wound closure, especially in postoperative meningitis (Huang et al., 2017, 2019).

Additionally, it must be considered that a small approach could lead to decreased illumination and overview in the surgical field during microsurgery (Caballero-García et al., 2021; Rodriguez Rubio et al., 2021); therefore, smaller approaches should be used selectively and should be based on the targeted pathology, such as for microvascular decompression and small and/or cystic tumors (Hoshide et al., 2018).

The median craniotomy area for non-oncological pathologies was smaller than that for oncological pathologies (634 mm^2^ vs. 776 mm^2^). The craniotomy area was also significantly and inversely related to the tumor volume. Thus, large tumors are operated upon using larger approaches. Tumor volume had a significant influence on the number of preoperative symptoms as well as on postoperative hemorrhage and hydrocephalus. Both complications significantly correlated with the occurrence and number of new permanent neurological deficits. Whether tumor volume influences postoperative CSF leakage remains controversial. VS is associated with hydrocephalus, which may remain asymptomatic before surgery. Thus, it is important to consider that hydrocephalus, and not only the size of the approach, could result in CSF leaks (Brennan et al., 2001; Gerganov et al., 2011; Hoffman, 1994). However, this relationship could not be confirmed in our study.

Although tumor volume also narrowly missed the significance of complete resection (GTR + Simpson I/II), a correlation may be assumed here, as statistical correlation was shown for meningiomas and VS. Considering the number of cases of the two entities combined (337/449; 75.1%), they accounted for most patients operated on. Another notable finding emerged in the case of VS. Here, a large tumor volume correlated with an increased incidence of postoperative meningitis. Huang et al. also noted this correlation. A possible reason may be the contamination of the CSF by bone dust, a higher incidence of CSF fistula due to the already existing hydrocephalus, or a longer operation time (Huang et al., 2019; Rahimpour et al., 2016).

Postoperative complications, hearing loss, and facial nerve palsy were the most associated pathologies. CSF fistulas are the third most common complication (Matthies and Samii, 1997; Sanna et al., 2004). In some studies, the incidence of CSF fistulas after surgery for VS via the RSA has been reported to be as high as 30% (Jun et al., 2020). A study by Sathaporntheera and Saetia reported an incidence of 14.0% in a mixed collection of 286 oncologic and non-oncologic patients. Our series showed a rate of 11.7% of the VS cases. The percentage of the total collective was 7.5%. The occurrence of CSF fistulas is frequently associated with further complications in the course (Sathaporntheera and Saetia, 2020).

Complications mentioned in the literature include wound-healing disorders, meningitis, and abscess formation. In some cases, revision surgery is necessary, which prolongs the in-patient stay and increases the risk of nosocomial infections. Temporarily, the patient's quality of life decreases (Alattar et al., 2017; Ha et al., 2016; Hutter et al., 2014; Kehler et al., 2012; Misra et al., 2009; Obaid et al., 2018).

Facial nerve palsy (FNP) is one of the most common postoperative complications. Hoshide et al. documented that 84% of patients had mostly mild postoperative facial nerve palsy. Immediately after surgery, a significantly higher proportion of patients suffer from temporary facial nerve palsy, which usually regresses during their in-patient stay (Hoshide et al., 2018). In our study, patients showed facial nerve palsy in 14.1% of cases, of which House-Brackmann grades II or III were present in 79.2%. In the case of VS, 30.3% of patients had postoperative new permanent facial nerve palsy, but 74.1% only had a House-Brackmann grade II or III, resulting in a satisfactory functional outcome.

Postoperative new hearing loss was found in 8.2% for the whole collective, and in 10.1% of patients with VS. Facial nerve palsy (30.3%) and hearing loss (25.5%) also represent the symptoms with the greatest proportion of postoperative deterioration in this study. Neither hearing loss nor facial nerve palsies, as new postoperative deficits, were associated with the occurrence of intraoperative or approach-related postoperative complications.

The optimal surgical strategy for achieving the best functional outcomes was discussed reluctantly (Gurgel et al., 2012). Since the evolution of microsurgical techniques and intraoperative neuromonitoring, complete tumor resection has become the ultimate goal (Matthies and Samii, 1997; Samii and Matthies, 1997). However, there is an ongoing trend towards minimizing postoperative malfunction by allowing tumor remnants and conducting adjuvant radiotherapy to preserve functionality (Goldbrunner et al., 2020). Complete surgical removal remains as the optimal treatment, but not at the cost of quality of life due to potentially avoidable postoperative neurological deficits (Samii and Gerganov, 2012), leading to what is known as function-preserving surgery. In most cases, the tumors treated in this study were benign, slow-growing, and slow-displacing tumors. Postoperative radiation and the close-watch-and-wait concept are important postoperative options. Such therapeutic modalities may be taken into consideration in accordance with and consultation with the patient to discuss possible options such as postoperative radiotherapy or revision surgery with maximum functional preservation as one of the options or an increased risk of injury to neurovascular structures with an attempt of maximum tumor removal.

In our study, complete resection was achieved in 57.2% of the oncological cases, while in the case of VS, only 49.9% achieved complete resection. In most cases, a conscious decision was made to leave the residual tumor capsule adherent to the brainstem or cranial nerves based on intraoperative neuromonitoring measurements. Capsule resection is often associated with higher rates of cranial nerve deficit. Moreover, the aggressive removal of epidermoid cysts may result in high postoperative morbidity if the cyst capsule afflicts crucial neurovascular structures (Kalani and Couldwell, 2018).

Gurgel et al. compared 30 studies on the removal of large VS. In most studies, the percentage of complete tumor resection ranged from 86% to 100% (Darwish et al., 2005; Gurgel et al., 2012; Misra et al., 2009; Samii et al., 2010; Yamakami et al., 2004). However, the estimated intraoperative EOR status was compared with the intraoperative MRI or CT in only 2 of the 30 studies. The GTR rates of these two studies were 42.7% (Zhao et al., 2010) and 30.6% (Lee et al., 2004), respectively.

It should be noted that several variables for complete tumor resection are independent of the surgical approach or the surgical skill and experience of the surgeon. These variables are well-described and include adhesion to the brainstem, involvement of nervous structures, invasion of the cavernous sinus, and tumor (Al-Mefty et al., 2020; Couldwell et al., 2007; Diluna and Bulsara, 2010; Heth and Al-Mefty, 2003; Natarajan et al., 2007a). Thus, a good functional outcome should be given the benefit of doubt over radical surgical tumor resection, as long as the tumor entity or benignity and growth characteristics permit.

After 43.2% of the procedures, patients in this study experienced at least one new permanent neurological deficit. Sekhar et al. also documented a similar overall rate of 47% in their study of petroclival pathologies. The overall rates are purely related to cranial nerve-related deficits (Natarajan et al., 2007a, 2007b). A comparison of the overall rate of new permanent deficits with other comparative studies is difficult, as many authors have summarized complications and only discussed single deficits. In this study, the occurrence or number of new permanent deficits showed no statistical correlation with the tumor volume or craniotomy area. However, the occurrence of postoperative CSF fistulas, hemorrhages, and hydrocephalus should be prevented as these are significantly related to new deficits.

Mastronardi et al. investigated the influence of patient age on the outcome after surgery via RSA for microvascular decompression in trigeminal neuralgia. However, they did not find any statistical correlation (Mastronardi et al., 2019). In the present study, patient age at the time of surgery did not play a significant role in the postoperative outcomes, improvement or worsening of preoperative neurological symptoms, postoperative KPSS score, nor occurrence of new permanent deficits. Although older patients are more prone to general postoperative complications after surgical procedures, other authors have not found increased rates of specific complications related to VS resection, such as postoperative cranial nerve deficits or CSF leaks (Helal et al., 2021; Luryi et al., 2020, 2021).

In the field of posterior skull base surgery, SVT is not an unrecognized complication but has rarely been addressed in the literature regarding RSA. In this study, a correlation was found between the preoperative presence of coagulation disorders and postoperative occurrence of SVT. Thrombophilia and increased bleeding tendency with intraoperative substitution may result in SVT; further prospective investigation is needed to distinguish these results. Kryskiewicz et al. did not find any correlations between them, but a correlation was found between the intraoperative sinus injury and the development of postoperative SVT (Krystkiewicz et al., 2022). However, this was not observed in our study. Although the presence of postoperative SVT was not statistically associated with the occurrence of new neurological deficits or deterioration of preoperative symptoms, SVT should be prevented. Therefore, intraoperative sinus injuries should be avoided at all costs.

Anatomical knowledge and hurdles in this region are the most important requirements to avoid unnecessary intra- and postoperative complications, which may jeopardize surgical success and satisfying postoperative outcomes.

### 4.1 Study limitations

As this was a retrospective case series, it was impossible to draw causalities concerning the clinical outcomes. Nevertheless, we implemented a detailed clinical examination in our clinical workflow, including functional performance scores and a standardized follow-up protocol based on a certified neuro-oncological board. However, the current study has some noteworthy limitations. In addition to its retrospective nature, the analyzed patient collective suffers from certain aspects of heterogeneity regarding pathology and tumor entities. We included all treated pathologies by focusing more on approach-related findings and less on oncological outcomes. With the aim of approach-related complications, we decided to focus not only on lesions but also on neurovascular compression syndromes due to the similarities in approach techniques; thus, complications could be reduced on the approaches as much as possible.

## 5 Conclusions

RSA must be performed with utmost care, and approach-related complications should not be neglected. Tumor size should not lead to more extensive approaches at any cost as postoperative complications increase. Individually tailored approaches are essential to obtain the best postoperative outcome, with precise tumor location and pathology in mind. Function-preserving surgery with postoperative radiotherapy or a close wait-and-watch approach may be a viable alternative, as GTR should not be performed.

## Funding

This study did not receive any specific grant from public, commercial, or non-profit funding agencies.

## Declarations of interest

Bernhard Meyer (BM) was a consultant for Brainlab (Brainlab AG, Feldkirchen, Germany). Additionally, BM works as a consultant and/or received personal fees and research grants from Medtronic, Spineart, Icotec, Relievant, Companion Spine, Medacta, Zeiss, Sonovum and Ulrich Medical, He has received personal fees and research grants from Medtronic, Ulrich Medical, BrainLab, Icotec, and Relievant for clinical studies. These are independent of the submitted work. Vicki M. Butenschoen is a consultant for Brainlab (Brainlab AG, Feldkirchen, Germany). There are no further conflicts of interest regarding the other authors.

## Data availability

All data generated or analyzed in this study can be provided upon reasonable request.

## Author contributions

Conceptualization: Amir Kaywan Aftahy; Formal analysis and investigation: Amir Kaywan Aftahy, Maria Goldberg, Vicki M. Butenschoen, Arthur Wagner; Writing – original draft preparation: Amir Kaywan Aftahy, Maria Goldberg; Writing – review and editing: Bernhard Meyer, Chiara Negwer; Supervision: Chiara Negwer.
